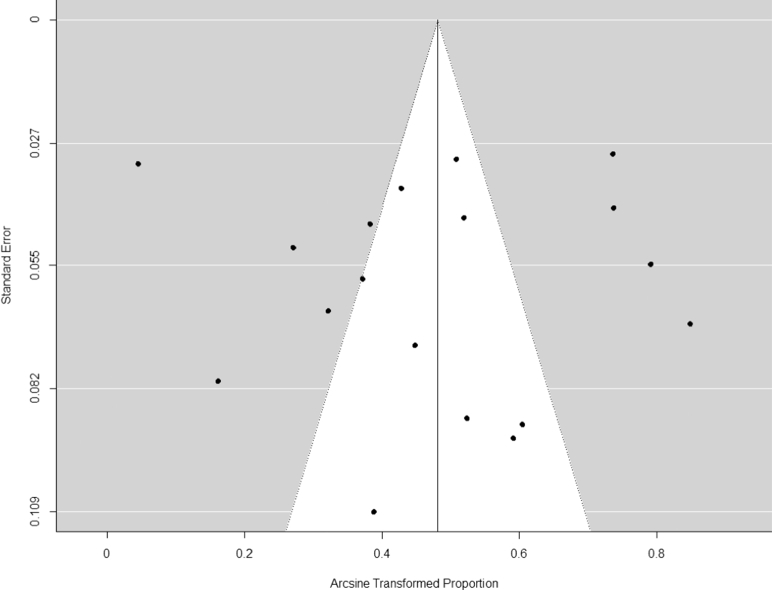# Systematic Review and Meta-analysis: Prevalence of Posttraumatic Stress Disorder in Trauma-Exposed Preschool-Aged Children

**DOI:** 10.1016/j.jaac.2021.05.026

**Published:** 2022-03

**Authors:** Francesca Woolgar, Harriet Garfield, Tim Dalgleish, Richard Meiser-Stedman

**Affiliations:** a University of East Anglia, Norwich, United Kingdom; b Addenbrooke’s Hospital, Cambridge, United Kingdom; c Cognition and Brain Sciences Unit, Medical Research Council, Cambridge, United Kingdom

**Keywords:** PTSD, trauma, preschool, children

## Abstract

**Objective:**

Trauma exposure is common in preschool-aged children. Understanding the psychological impact of such exposure and the prevalence of posttraumatic stress disorder (PTSD) in this population is important for provision of appropriate and timely intervention. This pre-registered (PROSPERO: CRD41019133984) systematic review and meta-analysis examined the prevalence of PTSD in trauma-exposed preschool-aged children.

**Method:**

Literature searches were conducted of PubMed (Medline), PsycINFO and PILOTS, alongside reference lists of relevant reviews. Studies were selected if they comprised trauma-exposed samples with a mean age of less than 6.5 years, and PTSD was assessed using standardized interviews at least 1-month post trauma. Information on sample characteristics, trauma exposure, PTSD measurement, and diagnostic criteria were extracted. For studies that applied more than one PTSD diagnostic algorithm, the most age-appropriate criteria were used to estimate pooled prevalence estimate across studies. A random-effects model was used for meta-analysis.

**Results:**

Eighteen studies were included (N = 1941). The pooled PTSD prevalence was 21.5% (95% CI = 13.8%-30.4%) when using the most developmentally appropriate diagnostic algorithm that was available. When focusing on the subset of studies that reported both standard adult criteria and age-appropriate criteria (k = 12), a pooled estimate of 4.9% (95% CI = 2.5%-8.0%) was obtained for standard adult criteria (*DSM-IV*), and 19.9% (95% CI = 12.1%-29.0%) was obtained for age-appropriate criteria (PTSD-AA). Prevalence was 3-fold higher following interpersonal and repeated trauma exposure, compared to non-interpersonal or single-event trauma, respectively. Higher prevalence was found when age-appropriate diagnostic tools were used. There was significant heterogeneity across studies and a lack of studies conducted in low-income countries and applying age-appropriate diagnostic algorithms.

**Conclusion:**

Preschool-aged children are vulnerable to developing PTSD following trauma exposure. Younger children show prevalence trends similar to those of older youths and adults following different types of trauma. Age-appropriate diagnostic criteria are essential to ensure that appropriate identification and early support are provided.

A large proportion of children experience trauma exposure before the age of 18 years.^1^^,^^2^ Copeland *et al.* found that more than two-thirds of children reported at least 1 traumatic event by age 16 years.^1^ Similarly, research conducted by Lewis *et al.* indicated that 31% of children had experienced trauma exposure before their 18^th^ birthday.^2^ Following exposure, some children naturally recover and show minimal signs of psychological distress.^3^ However, for a proportion of children, trauma exposure can result in longer-term debilitating psychological reactions, such as posttraumatic stress disorder (PTSD).^4^

For preschool-aged children (those up to 6 years of age), trauma exposure is unfortunately common. However, there are very few estimates of trauma exposure for this population. One survey that looked at interpersonal violence exposure reported that up to 44% of 2- to 5-year-olds have been exposed to at least 1 physical assault.^5^ However, as this survey did not include other types of trauma exposure, the prevalence of exposure to trauma in the early years is likely to be higher than this estimate. Furthermore, there is extensive variability in methodology and trauma definitions, thereby preventing a single prevalence figure to characterize trauma exposure in preschool-aged children. Despite the fact that younger children have had relatively less time to experience traumatic events, these events may be appraised in a more life-threatening way than in older children.^6^ In fact, research has suggested that following direct trauma exposure, young children develop PTSD at the same, or a higher, rate than older children and adults.^7^ Understanding what proportion of preschool-aged children are exposed to trauma and subsequently develop PTSD is critical for planning services, staff training, and screening efforts.

The assessment of PTSD in preschool-aged children is greatly complicated by their level of cognitive development, and the consequent reliance on caregiver report.^8^ Moreover, despite attempts to produce child-appropriate diagnostic criteria for PTSD^9^ within the *DSM-IV,* there are systematic differences in symptom manifestation in very young children compared to adults and older youths.^10^ The development of an age-appropriate alternative algorithm for the diagnosis of PTSD in pre-schoolers (PTSD-AA) revolutionized clinical assessment and service delivery.^10^ The PTSD-AA continued to be refined based on empirical findings,^11^^,^^12^ culminating in a new preschool-subtype of PTSD in the *DSM-5:* posttraumatic stress disorder for children 6 years and younger (*DSM-5* PTSD<6Y).^13^ Now that these revised age-appropriate diagnostic criteria are well established, there is a critical mass of relevant studies to support a meta-analytic synthesis to estimate the prevalence of PTSD in young children.

The use of different diagnostic algorithms has had an impact on prevalence estimates in older children and adults.^14^ Other factors that have an impact on prevalence estimates include the type of assessment measure^15^ and the informant.^16^ Importantly, the type of trauma also has a large impact on estimated rates and trajectories of PTSD in children and adults.^4^^,^^17^ Rates of PTSD in children and adolescents are higher following interpersonal trauma compared to non-interpersonal trauma,^1^^,^^4^^,^^18^ and exposure to intentional or assaultive injury is associated with higher rates of PTSD in both the acute phase and longer term.^19^ It is clear that there are many factors that influence PTSD prevalence rates across different populations. Therefore, it is important to assess the influence of these factors on PTSD prevalence rates in preschool-aged children.

### Current Meta-analysis

The aim of this meta-analysis is therefore to understand the prevalence of PTSD in young children aged 0 to 6 years who have directly experienced a traumatic event. We conducted sensitivity analyses to examine the effects on prevalence of applying the different diagnostic algorithms (eg, PTSD-AA; *DSM-IV*) in this population.

Previous meta-analyses of children and adolescents report high levels of heterogeneity across samples,^4^^,^^20^ potentially as a function of different types of trauma exposure. We therefore used moderator analyses to explore the influence of trauma exposure characteristics outlined above (eg interpersonal vs non-interpersonal trauma^1^^,^^4^^,^^18^) on the prevalence of PTSD in young children.

Understanding the prevalence of PTSD in young children, and elucidating the possible factors that may have an impact on prevalence, will facilitate professionals in better identifying and supporting young children who may be vulnerable following a trauma.

## Method

This review was pre-registered on PROSPERO (CRD41019133984).

### Selection of Studies

Relevant studies were identified through systematic searches in 3 electronic databases: PubMed(Medline), PsycINFO, and the Published International Literature on Traumatic Stress (PILOTS). Relevant papers were also obtained from the reference list of a recent review in the field.^14^ Searches were restricted to empirical English-language papers published in peer-reviewed journals between 1980 (when PTSD was first introduced in the *DSM-III*^21^) and July 10, 2019. Poster abstracts and unpublished studies were excluded. Medical Subject Headings (MeSH) terms were applied to the searches for PsycINFO and PubMed (Medline). Non-MeSH terms were searched within the title or abstract: (((MeSH CHILD, PRESCHOOL) OR (MeSH Infant)) OR (Toddler∗ OR preschool∗ OR child∗)) AND ((MeSH Stress Disorders, Post-traumatic) OR (PTSD OR “post-traumatic stress disorder” OR “posttraumatic stress disorder” OR “post traumatic stress disorder”)). The following search terms were applied to the PILOTS database: (Toddler∗ OR preschool∗ OR child∗) AND (“PTSD” OR “post-traumatic stress disorder” OR “posttraumatic stress disorder” OR “post traumatic stress disorder”).

### Inclusion and Exclusion Criteria

The following eligibility criteria were used:

1) Participants were all directly exposed to trauma as defined by the *DSM-5* Criterion A for PTSD. Samples of children who had only indirect exposure (eg, hearing about the event via media reports) were excluded.
2) Participants were identified on the basis of being trauma exposed. Studies were excluded if participants were recruited because they had posttraumatic stress symptoms and/or they were seeking psychological treatment.
3) The study population needed to include preschool-aged children 6 years of age or less. If the age range exceeded 6 years, then studies were included if the mean sample age was less than 6.5 years.
4) The study assessed PTSD diagnoses and symptoms using a structured clinical interview at least 1 month after the trauma.
5) The record provided enough information to derive the prevalence of PTSD in the sample.

**Figure 1:**
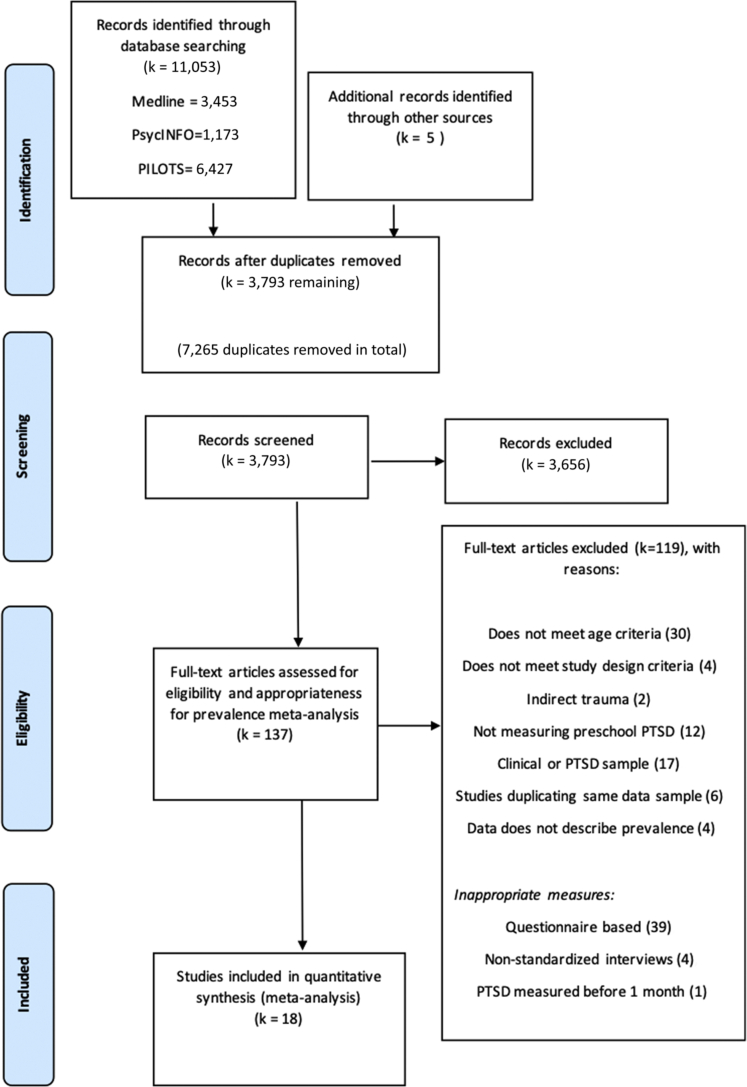
PRISMA Diagram Outlining the Search and Exclusion Process ***Note:****PILOTS = Published International Literature on Traumatic Stress. Excluded articles with justifications can be found in*Supplement 2*, available online.*

Screening and selection of studies was conducted by the first author. Eighteen studies met all eligibility criteria (a Preferred Reporting Items for Systematic Reviews and Meta-Analyses [PRISMA] flowchart is provided in Figure 1).

### Data Extraction

Information on the sample characteristics (size, age range, mean age and standard deviation (SD), proportion of male participants, country), nature of trauma exposure (categorized as group vs individual, interpersonal (war, terrorism, interpersonal violence) versus non-interpersonal (accidental trauma or medical illness), single-event exposure versus repeated exposure to the trauma), measurement of PTSD (interview used, time since trauma, informant), diagnostic criteria algorithm applied (*DSM-IV, DSM-5* PTSD<6Y, or PTSD-AA), and outcomes of the PTSD assessment were extracted from eligible studies by the first author. The data extracted from all studies was checked by 2 authors (RMS, HG). Differences with respect to study classification were resolved by FW and RMS.

### Quality of Studies

The quality of each study was assessed using a risk of bias tool adapted from the Joanna Briggs Institute (Prevalence Critical Appraisal Tool).^22^ The risk of bias assessment tool comprised 6 questions and assessed the quality and representativeness of the sample, nonresponse rates and reasons, recruitment procedures, and inclusion and exclusion criteria (Supplement 1, available online). Each study was allocated a risk-of-bias rating by the first author (9−12 = low risk of bias, 5−8 = medium risk, 0−4 = high risk). All studies were also rated by the second author (HG). The Cohen kappa showed that there was moderate interrater agreement between the 2 ratings (κ = 0.54, *p* < .001). Discrepancies were discussed and resolved. Study ratings for each risk-of-bias criterion are detailed in Table S1, available online.

### Statistical Analysis

Analyses were performed using the metafor^23^ package in R. The prevalence of preschool-aged children who reached the threshold for PTSD was extracted from each paper. For those papers that included multiple prevalence data using different diagnostic algorithms, the best available data from the most developmentally appropriate diagnostic algorithm was used to calculate the main pooled prevalence estimate. So, for example, if a study reported prevalence estimates according to both PTSD-AA and *DSM-IV* PTSD algorithms, then the PTSD-AA would be the study’s most developmentally appropriate algorithm and would be used in the pooled prevalence calculations. However, if a study used only the *DSM-IV* algorithm, then this would be the study’s most developmentally appropriate algorithm. Each study’s most age-appropriate diagnostic algorithm was referred to as the study’s “optimal” criteria. Table 1 illustrates the differences among the 3 diagnostic algorithms and includes comments on their age-appropriateness, therefore providing a hierarchy to establish each study’s “optimal” criteria. As only 3 studies^24^, ^25^, ^26^ used the proposed *DSM-5* PTSD<6Y criteria (and also reported PTSD-AA criteria), the “optimal” study criteria used in the pooled prevalence were either the PTSD-AA or the *DSM-IV* criteria. However, a further study^27^ used another algorithm (3 or more re-experiencing, 1 or more avoidance, 2 or more hyperarousal) that could not be classified as either PTSD-AA or *DSM-IV*; this study was included in the “optimal” algorithm meta-analysis and moderator analyses, but was not considered in further analyses that considered prevalence estimates for either *DSM-IV* or PTSD-AA algorithms. A random effects model was then used to compute a weighted estimate of the prevalence of PTSD. The arcsine transformation was used to account for issues with study weightings (eg, 95% CIs going below zero).^28^^,^^29^

**Table 1:** Hierarchy of “Optimal” Diagnostic Criteria

Diagnosis (Year)	Notes	Criteria
*DSM-5* PTSD<6Y (2013)	Incorporates changes in PTSD-AA. Takes into account developmental age. Increased focus on behavioral symptoms, rather than thoughts and feelings.	Criterion A: 1) Direct experience of trauma, 2) Witnessing person experience trauma, 3) Learning traumatic event occurred to parent or care-giver Criterion B: Intrusion symptoms (1 or more symptoms) Criterion C: Persistent avoidance (1 or more symptoms) Criterion D: Negative alterations in cognitions and mood (2 or more symptoms) Criterion E: Alterations in arousal and reactivity (2 or more symptoms) Criterion F: Persistence of symptoms for more than 1 month Criterion G: Significant symptom-related distress or functional impairment
PTSD-AA (1995)	Advance over *DSM-IV,* to make diagnostic criteria more age-appropriate. Takes into account developmental age. Focus on behavioral symptoms, rather than thoughts and feelings.	Criterion A: The person experienced, witnessed, or was confronted with an event or events that involved actual or threatened death or serious injury, or a threat to the physical integrity of self or others. Note: extreme reaction at time of the event is not required. Criterion B: Intrusion symptoms (1 or more symptoms) Criterion C: Persistent avoidance (1 or more symptoms) Criterion D: Increased arousal (2 or more symptoms) Criterion E: Persistence of symptoms for more than 1 month Criterion F: Significant symptom-related distress or functional impairment
*DSM-IV* (1994)	Based on research in adults and older children. Symptoms are not appropriate for young children’s developmental level, eg, verbal expression, memory, and abstract thought.	Criterion A: 1) The person experienced, witnessed, or was confronted with an event or events that involved actual or threatened death or serious injury, or a threat to the physical integrity of self or others 2) the person’s response involved intense fear, helplessness, or horror. Note: In children, this may be expressed instead by disorganized or agitated behavior. Criterion B: Intrusion symptoms (1 or more symptoms) Criterion C: Persistent avoidance (3 or more symptoms) Criterion D: Increased arousal (2 or more symptoms) Criterion E: Persistence of symptoms for more than 1 month Criterion F: Significant symptom-related distress or functional impairment

Heterogeneity of studies was assessed by the Cochran Q test^30^ and the I^2^ statistic.^31^ An I^2^ between 30% and 60% indicates moderate heterogeneity, between 50% and 90% substantial heterogeneity, and ≥75% considerable heterogeneity.^32^ Potential publication bias was assessed through inspection of funnel plots and the Egger test for funnel plot asymmetry.^33^

A sensitivity analysis considered how prevalence varied as a function of the different diagnostic algorithms for PTSD (*DSM-IV* and PTSD-AA) for the subgroup of studies that used both the PTSD-AA and *DSM-IV.*

Moderator analyses using random effects models were run to identify differences in prevalence due to different types of trauma (interpersonal vs non-interpersonal, group vs individual, and single-event vs repeated). The Holm−Bonferroni method^34^ was used to correct for multiple comparisons. We also examined the effect of study quality (low risk of bias vs medium−high risk of bias). Other potential moderator questions (eg, effects of demographic variables such as sex) were precluded because of insufficient numbers of studies providing relevant data.

## Results

In total, 18 study records were included in the meta-analysis, comprising 1,941 trauma-exposed young children (study samples ranged in size from 21 to 284) (PRISMA flowchart in Figure 1).

### Characteristics of Studies

The characteristics of the included studies are shown in Table 2.^35^, ^36^, ^37^, ^38^, ^39^, ^40^, ^41^, ^42^, ^43^, ^44^, ^45^, ^46^, ^47^, ^48^ Participants ranged in age from 0 to 16 years. Three studies included children more than 6 years of age, but had a total mean age of less than 6.5 years. The estimated mean age across all studies was 4.5 years (4 studies did not report mean age). Approximately 56% of participants were male (2 studies did not report sex). Different types of trauma were reported as follows: interpersonal trauma (k = 8), non-interpersonal trauma (k = 9), single-event trauma (k = 10), repeated trauma (k = 7), group trauma (k = 5), and individual trauma (k = 12). One study^26^ collated prevalence for a mix of traumas (interpersonal, non-interpersonal, individual, group, repeated and single-event).

**Table 2:** Studies Included in the Meta-analysis

Authors, Reference	Year	Type of trauma	Single/repeat	Interpersonal/non-interpersonal	Individual /group	Age range (mean, SD)	Ethnicity (%)	N	Proportion males (%)	Time point (mo)	Measure	Optimal diagnostic criteria	Risk of bias category (/12)
Cohen *et al.*^35^	2009	Terrorism	Repeat	Interpers.	Grp–	3.5-7.5 (5.47 y, 1.34)	Isr (100)	29	70	6-18	PTSDSSI	PTSD-AA	High (4)
De Young *et al.*^24^	2011	Accidental trauma	Single	Non-interpers.	Ind–	1-6 (2.7 y, 1.54)	DNR	130	52	1	DIPA	PTSD-AA	Low (12)
DeVoe *et al.*^27^	2006	Terrorism	Single	Interpers	Grp–	0-5 (DNR)	Wh (71), Bl/Hi (6), Mx (17), Oth (6)	180	NR	9-12	PTSDSSI	Other^f^	Med (6)
Gigengack *et al.*^25^	2015	Accidental trauma	Single	Non-interpers	Ind–	0-7 (6.2 y, 2.7)	DNR	98	68	26^a^	DIPA	PTSD-AA	Low (10)
Graf *et al.*^36^	2011	Accidental trauma	Single	Non-interpers	Ind–	1-4 (32 mo, 9.5)	DNR	76	58	15^a^	PTSDSSI	PTSD-AA	Low (11)
Graf *et al.*^37^	2013	Medical illness	Single	Non-interpers	Ind–	0-4 (34.8 mo, 11)	DNR	48	65	15^a^	PTSDSSI	PTSD-AA	Low (9)
Graham-Bermann *et al.*^38^	2012	IPV	Repeat	Interpers	Ind–	4-6 (4.93 y, 0.86)	Lat (5), Af Am (37), EuAm (38), Mx (20)	85	53	<24	PTSDSSI	PTSD-AA	High (3)
Koolick *et al.*^39^	2016	IPV	Repeat	Interpers	Ind–	4-6 (4.96y, 0.815)	Wh (33), Lat (19), Af Am (31), Mx (17)	144	52	<24	PTSDSSI	PTSD-AA	Med (5)
Meiser-Stedman *et al.*^40^	2008	Accidental trauma	Single	Non-interpers	Ind–	2-6 (DNR)	Wh (45), Oth (55)	60	53	6^b^	PTSDSSI	PTSD-AA	Low (11)
Modrowski *et al.*^41^	2013	IPV	Repeat	Interpers	Ind–	4-6 y (5.0 y, 0.93)	Hi (7), Af Am (24), Eu Am (45), Mx (24)	55	NR	<24	PTSDSSI	PTSD-AA	Med (5)
Ohmi *et al.*^42^	2002	Accidental trauma	Single	Non-interpers	Grp–	1-3 y (DNR)	Jap (100)	32	66	6	CPTSD-RI	PTSD-AA	Low (11)
Pat-Horenczyk *et al.*^43^	2013	War	Repeat	Interpers	Grp	DNR (Mixed^c^)	DNR	262	61	Mixed^d^	PTSDSSI	*DSM-IV*	High (4)
Scheeringa *et al.*^26^	2012	Mixed	Mixed	Mixed	Mixed–	3-6 y (Mixed^e^)	Wh (21), Af Am (67), Mx (8), Oth (4)	284	62	NR	PAPA	PTSD-AA	Med (6)
Scheeringa *et al.*^44^	2006	Accidental trauma	Single	Non-interpers	Ind–	0-6 y (DNR)	Bl (43), Unk (57)	21	67	2	PTSDSSI	PTSD-AA	Low (10)
Stoddard *et al.*^45^	2017	Accidental trauma	Single	Non-interpers	Ind–	1-4 y (1.93 y, DNR)	Wh (67), Bl (10), Hi (14), As (2), Mx (5), Oth (2)	39	57	1	DICA-P and PTSDSSI	PTSD-AA	Low (10)
Swartz *et al.*^46^	2011	IPV	Repeat	Interpers	Ind–	4-6 y (63.8 mo, 11.2)	Hi (6), Af Am (29), Eu Am (46), Mx (20)	34	54	<24	PTSDSSI	PTSD-AA	High (2)
Viner *et al.*^47^	2012	Medical illness	Single	Non-interpers	Ind–	3-16 y (6.5 y, 2.8)	Wh (92), Bl (<1), As (2), Mx/Oth (5)	245	42	>36	DAWBA	*DSM-IV*	Med (8)
Wolmer *et al.*^48^	2015	War	Repeat	Interpers	Grp–	3-6 (64.12 mo, 8.48)	DNR	122	50	>3	PTSDSSI	PTSD-AA	High (4)

Note: Af Am = African American; As = Asian; Bl = Black; CPTSD-RI = Childhood PTSD Reaction Index; DAWBA = Development and Well-Being Assessment; DICA-P = Diagnostic Interview for Children and Adolescents; DIPA = Diagnostic Infant and Preschool Assessment; DNR = did not report; Eu Am = European American; Hi = Hispanic; IPV = interpersonal violence; Isr = Israeli; Jap = Japanese; Lat = Latino/a; Mx = mixed; Oth = other; PAPA = Preschool Age Psychiatric Assessment; PTSD-AA = PTSD Alternative Algorithm; PTSDSSI = PTSD Semi-Structured Interview; SSIORIYC = Semi-Structured Interview and Observational Record for Infants and Young Children; Unk = unknown; Wh = White.

a Average time since trauma.

b 2- to 4-Week data also reported, but not included in meta-analysis.

c Continuous sample (mean age = 3.00 y, SD = 1.44), past sample (mean age = 3.44 y, SD = 1.33).

d Ongoing trauma or past trauma (time since trauma not recorded).

e Single event (mean age = 5.2 y, SD = 1.1), Hurricane Katrina (mean age = 5.1 y, SD = 1.0), repeated trauma (mean age = 5.1 y, SD = 1.1).

f Diagnostic algorithm using criteria of 3 or more re-experiencing, 1 or more avoidance, and 2 or more hyperarousal symptoms.

Thirteen studies used the Posttraumatic Stress Disorder Semi-Structured Interview (PTSDSSI)^10^^,^^12^ to assess PTSD prevalence. Other studies used the Diagnostic Infant and Preschool Assessment (DIPA; k = 2),^49^ the Development and Well-Being Assessment (DAWBA; k = 1),^50^ the Preschool Age Psychiatric Assessment (PAPA; k = 1),^51^ the Childhood PTSD Reaction Index (CPTSD-RI; k = 1),^52^ and the Diagnostic Interview for Children and Adolescents (DICA-P; k = 1).^45^ Some studies used more than one PTSD diagnostic algorithm. Fifteen studies used the PTSD-AA algorithm to assess PTSD, and 14 studies reported prevalence using the *DSM-IV.* Twelve studies compared prevalence of PTSD when using the PTSD-AA and the *DSM-IV.* Three of these studies also compared prevalence when using the proposed algorithm for the *DSM-5* PTSD<6Y; however, these studies gathered their data before the *DSM-5* PTSD<6Y was published, and because they also reported on the PTSD-AA, the latter prevalence estimates were used. One study^27^ used an alternative algorithm (3 or more re-experiencing, 1 or more avoidance, 2 or more hyperarousal).

One paper compared the prevalence between therapists and caregivers as informants.^32^ The prevalence from the therapists was not included in this meta-analysis, because of all other studies using only caregivers as informants. The studies varied in time-since-trauma. One study reported prevalence at 2 to 4 weeks and 6 months post trauma.^40^ For the purpose of this meta-analysis, and in line with the exclusion criteria, only the 6-month follow-up data were included. As such, time since trauma ranged from 1 month to 3 years across studies. Reported prevalence of PTSD ranged from 0% to 65%.

### Pooled Prevalence Estimate

**Figure 2:**
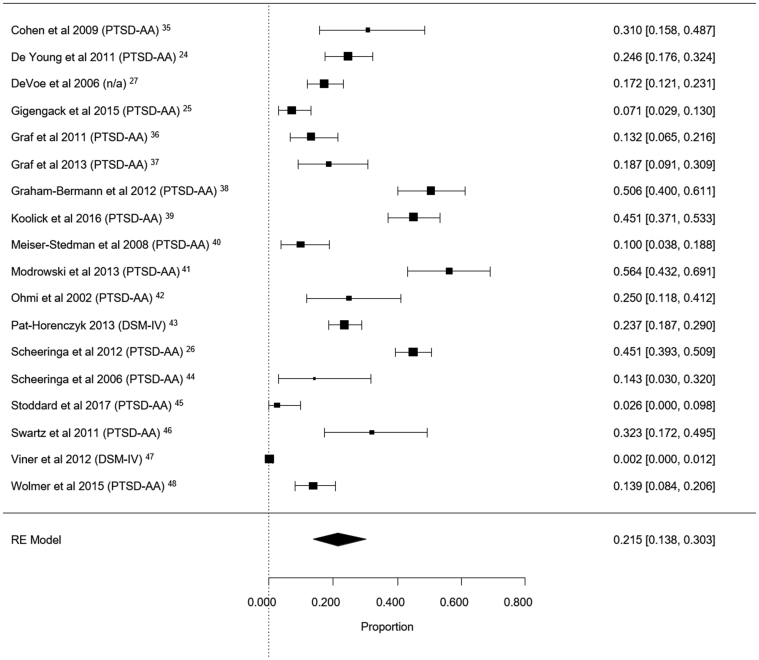
Forest Plot for Overall Prevalence Levels Using Optimal Diagnostic Criteria Applied in Each Study ***Note:****This figure gives proportions rather than percentage prevalence (ie, multiply by 100 for percentage estimates. RE = random effects, study-specific odds ratios (95% CIs) are denoted by black boxes (black lines) and presented in the right-hand column. The combined proportion estimate for all studies is represented by a black diamond, where diamond width corresponds to 95% CI bounds. Box and diamond heights are inversely proportional to the precision of the proportion estimate.*

Prevalence levels according to each study’s “optimal” diagnostic criteria were used to derive a pooled prevalence estimate of PTSD prevalence in young children of 21.5% (95% CI 13.8-30.4%) (for forest plot, see Figure 2). The Q test result was significant (Q = 416.81, df = 17; *p* < .001), indicating considerable heterogeneity between studies (I^2^ = 94.9). However, given the differences in study diagnostic systems, this may misrepresent prevalence. We therefore undertook sensitivity analyses to look at the impact that different diagnostic algorithms had on prevalence estimates (see below).

A pooled prevalence was also estimated using the subsample of studies that included only children aged 6 years and younger (k = 15). The pooled prevalence estimate of PTSD in this subsample was 24.8% (95% CI = 16.9%−33.7%). The Q test result was significant (Q = 175.93, df = 14; *p* < .001), indicating considerable heterogeneity between studies (I^2^ = 93.0).

Other prevalence levels reported, but not used in this meta-analysis, were as follows. Three studies using the proposed *DSM-5* PTSD<6Y criteria prior to the *DSM-5* publication (in addition to *DSM-IV* and PTSD-AA) produced a combined prevalence of 23.9% (95% CI = 6.2%−48.3%).^24^, ^25^, ^26^ Another used the *DSM-IV* with therapists as respondents and yielded a prevalence of 22.0%.^41^ A final study using the PTSD-AA at a second time point (6 months) reported a PTSD prevalence of 10.0%.^24^

### Sensitivity Analysis

Prevalence levels were compared using the subgroup of 12 studies that reported prevalence using both the *DSM-IV* and PTSD-AA (Table 3). This sensitivity analysis indicated that the prevalence was higher when the PTSD-AA diagnostic criteria (19.9%) were used compared to when the *DSM-IV* criteria (4.9%) were used.

**Table 3:** Meta-analysis Outcomes for Prevalence, Including Moderator and Sensitivity Analyses

	K	N	Prevalence (%)	95% CI		Heterogeneity	
				Lower	Upper	Q test	I^2^
All studies using best available algorithm	18	1,941	21.5	13.8	30.4	416.81	94.9
Under 6 years subset	15	1,569	24.8	16.9	33.7	175.93	943.0
**Moderator analyses**							
**Moderator: PTSD-AA vs *DSM-IV* for optimal criteria applied**							
PTSD-AA criteria	15	1,254	24.3	16.0	33.8	193.56	92.4
*DSM-IV* criteria	2	507	7.5	0.0	44.5	108.76	99.1
Comparison	QM (df = 1) = 2.25, *p* = .1338^a^						
**Moderator: Interpersonal vs non-interpersonal**							
Interpersonal trauma	8	908	32.6	21.9	44.4	85.60	92.0
Non-interpersonal trauma	9	749	10.7	4.9	18.4	103.09	88.0
Comparison	QM (df = 1) = 10.83, *p* = .0010^a^						
**Moderator: Group vs individual trauma**							
Group trauma	5	625	20.3	15.4	25.7	8.52^b^	53.1
Individual trauma	12	1,032	19.6	9.6	32.2	315.13	95.2
Comparison	QM (df = 1) = 0.04, *p* = .8370^a^						
**Moderator: Single vs repeated trauma**							
Single event	10	929	11.3	5.8	18.4	116.90	88.4
Repeated trauma	7	736	35.3	23.7	47.8	68.81	90.6
Comparison	QM (df = 1) = 12.84, *p* = .0003^a^						
**Moderator: High vs low quality**							
High quality	8	504	13.5	8.1	19.9	26.57	72.1
Low quality	10	1,437	28.6	16.1	43.1	367.16	96.9
Comparison	QM (df = 1) = 3.68, *p* = .0551^a^						
**Sensitivity analyses:**							
PTSD-AA vs *DSM-IV* compared within studies							
PTSD-AA	12	1,024	19.9	12.1	29.0	150.98	91.0
*DSM-IV*	12	1,027	4.9	2.5	8.0	48.10	73.9
PTSD-AA vs *DSM-IV,* whole sample							
PTSD-AA	15	1,254	24.3	16.0	33.8	193.56	92.4
*DSM-IV*	14	1,534	5.2	2.3	9.1	158.30	87.9

Note: PTSD-AA = PTSD−alternative algorithm.

a Indicates the overall comparison of the two variables.

b Q test was nonsignificant at *p* < .05.

### Moderator Analysis

Moderator analyses were conducted to look at differences in prevalence following interpersonal or non-interpersonal trauma, group or individual trauma, and single or repeated trauma, using Holm−Bonferroni corrected alpha values. One study^26^ was excluded because the prevalence reported included a mix of these moderator variables. Moderator analyses therefore involved 17 studies.

The PTSD prevalence was higher following exposure to interpersonal trauma (32.6%) compared to non-interpersonal trauma (10.7%; *p* = .0010). Prevalence was significantly higher following repeated traumas (35.3%) compared to single traumas (11.3%; *p* = .0003). No significant difference was found for group trauma compared to individual trauma (*p* = .837).

A further moderator analysis found no significant impact of study quality on prevalence (*p* = .055); the prevalence estimate for studies with medium/high risk of bias was 28.6%, and was 13.5% for studies with low risk of bias.

### Publication Bias

Inspection of a funnel plot (see Figure S1, available online) and Egger test for funnel plot asymmetry (*z* = 0.047, *p* = .963) suggested no evidence that publication bias was skewing the prevalence estimate.

## Discussion

This meta-analysis investigated the prevalence of PTSD in preschool-aged children directly exposed to a traumatic event. Our findings estimate that around one-fifth of exposed children meet criteria for PTSD. Importantly, a pooled prevalence estimate of the subsample of papers in which only children aged 6 years and younger were included provided a prevalence estimate of 24.8%. Therefore, even though this meta-analysis included 3 studies that included children more than 6 years of age (although all study samples had a mean age of less than 6.5 years), it seems that the overall pooled prevalence estimates were not being artificially increased by the inclusion of older children. These prevalence rates exceed meta-analytic estimates for older children and adolescents (16%^4^). There was a nonsignificant relationship between study quality (ie, risk of bias) and prevalence, suggesting that our prevalence estimate was not biased by poor-quality studies; however, it is important to note that lack of studies may have mitigated our ability to detect any such effect.

The majority of included studies applied an age-appropriate diagnostic algorithm for PTSD (PTSD-AA), but 2 studies used only the adult-derived *DSM-IV* criteria. Moderator analyses did not find a higher prevalence for those studies that used PTSD-AA (24.3%) compared to the *DSM-IV* (7.5%; *p* = .1338), but this is likely due to the small number of studies that considered only *DSM-IV.* A follow-up sensitivity analysis focusing on those studies that used both *DSM-IV* and PTSD-AA algorithms indicated that prevalence was considerably lower when the *DSM-IV* criteria were adopted (4.9%) compared to the PTSD-AA criteria (19.9%). This finding corroborates previous findings^26^^,^^53^ suggesting that the *DSM-IV* criteria detect fewer cases of PTSD in this young population. Furthermore, despite the fact that the PTSD-AA requires fewer endorsed symptoms compared to the *DSM-IV,* no difference in symptom counts have been found in children who meet the *DSM-IV* or the PTSD-AA diagnostic criteria for PTSD.^11^^,^^40^ There is therefore no support for the higher prevalence rates of PTSD based on the PTSD-AA being due to the lower number of required symptoms. The PTSD-AA was developed to focus on more developmentally appropriate symptoms of PTSD, in particular, on behavioral symptoms that are easier for others to observe and therefore to report. The present finding therefore emphasizes the need for researchers and clinicians to apply age-appropriate diagnostic criteria to ensure that PTSD in vulnerable children does not go undiagnosed.

Statistically significant relationships between trauma-exposure type and PTSD prevalence were found. The repeated versus single-event trauma (35.3% vs 11.3%) prevalence contrast in preschool-aged children was pronounced, and is consistent with the adult literature.^54^^,^^55^ Similarly, exposure to an interpersonal trauma resulted in a trebling of prevalence relative to non-interpersonal trauma (32.6% vs 10.7%), a finding consistent with research showing that interpersonal trauma leads to greater psychological difficulties in older children and adolescents.^4^ No significant difference was found following individual trauma compared to group trauma. It is important to note that the number of available studies for these moderation analyses was limited, thereby reducing the available statistical power. Furthermore, even when accounting for trauma type, the level of heterogeneity between studies remained significantly high.

The current findings suggest that a significant minority of preschool-aged children meet criteria for PTSD following direct exposure to a traumatic event. It was previously thought that young children lacked the requisite cognitive capacity and maturity, such as the level of memory development or an understanding of the inherent dangers in trauma, to develop PTSD.^56^ However, this meta-analysis, in summarizing a literature that has emerged over the past 2 decades, indicates that these assumptions were misplaced. Clinicians, and the care systems around young children, therefore need to be aware of the potential psychological impacts of trauma exposure on this age group. Furthermore, it is important that clinicians be aware of the high risk of vulnerability in this young population following interpersonal and repeated trauma exposure. Relatedly, having an insight into the relatively high prevalence of PTSD in young children following trauma exposure, alongside the possible factors that might moderate a young child’s likelihood of developing PTSD should assist clinicians in remaining appropriately alert to this clinical presentation and in providing mental health support to those in need.

A key outcome, which is directly relevant to clinical practice, is corroboration of the need to use age-appropriate diagnostic criteria when assessing preschool-aged children for PTSD, to ensure that children are not overlooked or their PTSD is not diagnosed and consequently not supported.

Future research must assess preschool-aged children using age-appropriate diagnostic tools to ensure that accurate prevalence is being reported. An increase in studies in this area will enable researchers to better examine putative moderator variables (such as type of trauma, sex, trauma history, etc) that may contribute to different prevalence estimates and thus help to identify those most at risk. Using data from different informants will also help to provide a better picture of the prevalence of PTSD in this age group.

There was high heterogeneity across studies included in the meta-analysis. This likely reflects the different types of trauma to which the samples were exposed, as well as other methodological features of each study such as population, country, and the specific PTSD interview used. Heterogeneity remained significantly high even when different types of traumas were compared in the moderator analyses. As such, firm conclusions regarding the prevalence of PTSD in preschool-aged children are not possible, even when considering specific trauma types. The majority of the studies included in our analysis were also rated as being at moderate to high risk of bias. A moderator analysis indicated no significant impact on prevalence estimates due to increased risk of bias.

All studies included in this meta-analysis were also English-language papers from Organisation for Economic Co-operation and Development countries, thereby limiting the generalizability of our findings and underlining the need for future research in low- and middle-income countries. In addition, although the studies that reported on the sample ethnicity included a diverse range of ethnicities, 6 papers did not report on their samples’ ethnicity. This therefore limited our understanding or ability to comment on the role of ethnicity in this meta-analysis.

All studies included in this review used caregiver reports in interviews. This is unavoidable because of the age of the target population, but it is important to consider the caregiver’s own psychological responses to their child’s trauma, which may have affected their reporting of their child’s symptoms. Research has shown that caregivers can underestimate the level of trauma exposure that a child has experienced, as well as their PTSD symptoms.^40^^,^^57^, ^58^, ^59^, ^60^ Indeed, a recent study found that self-reported parent distress post trauma was the strongest correlate of child PTSD symptoms up to 3 years later.^61^

Finally, although this study estimated that around 22% of trauma-exposed preschool-aged children met criteria for PTSD, we do not know whether this prevalence estimate is stable across the whole age range (0−6 years). Because of the limited number of available studies in preschool-aged children, fine-grained prevalence estimates across the age range are currently not possible. Furthermore, viable moderator analyses were limited by the small amount of available studies; for example, ideally we would have further broken down the trauma type analyses to compare, for instance, accidental versus non-accidental traumas.

In summary, almost one-fourth of trauma-exposed preschool-aged children meet diagnostic criteria for PTSD. Younger children show similar variations in prevalence levels as a function of different types of trauma exposure (interpersonal vs personal; single vs repeated trauma) to older children, adolescents, and adults. Individuals in support systems around young children need to be aware of the psychological impact that trauma exposure can have on this population. Age-appropriate diagnostic criteria for diagnosing PTSD in this age group should be mandated to ensure appropriate identification and early support.

## Figures and Tables

**Figure S1:**