# Supplementary material for: Systematic Review and Meta-analysis: Prevalence of Posttraumatic Stress Disorder in Trauma-Exposed Preschool-Aged Children
Source: J Am Acad Child Adolesc Psychiatry. 2022 Mar;61(3):366–77. doi: 10.1016/j.jaac.2021.05.026 (PMC8885427; doi:10.1016/j.jaac.2021.05.026)
Supplement: Supplement 1 [file mmc1.docx]

**Supplement 1**

**Quality Checklist for Prevalence Meta-Analysis**

**Checklist to assess each study’s quality.**

*Score 0, 1 or 2 for each question on each study.*

**Assessed by: ______________**

**Population**

***Were participants and setting well described?***

(2)Information regarding the characteristics (age, gender, ethnicity) of the sample and trauma variables (type, severity, duration) are well described with the setting well reported (health setting, country, geography)

(1)Some information regarding participants characteristics and trauma variables are reported, with limited information on the setting

(0)Sample characteristics, trauma variables and setting information are not reported in any detail

***Was participation rate of those eligible at least 50%?***

(2)More than 50% of those eligible to participate took part

(1)Less than 50% of those eligible to participate took part

(0)The number of eligible potential participants was not reported

***Were reasons for non-response described?***

(2)Reasons for non-response were described with the number of those participants not responding reported

(1)Reasons were described for non-responders but no numbers provided OR Numbers of non-responders are reported but with no reasons

(0)Non-response rates were not reported in the study

***Was the sample representative – were there differences between those participants taking part and those not?***

(2)There were no significant differences in demographics or trauma variables between those participating and those not

(1)Reported significant differences between those participating and those not

(0)Differences between participants and those not taking part were not reported

***Were participants recruited in an appropriate way?***

(2)Consecutive or random sampling was used to recruit potential participants in person by the research team

(1)Consecutive or random sampling was used to recruit potential participants via letter or phone call

(0)Recruitment procedures were not reported in the study

***Were inclusion and exclusion criteria explicit and appropriate?***

(2)Inclusion and exclusion criteria were reported in detail

(0)Inclusion and exclusion criteria were not reported
