# Supplementary material for: Systematic Review and Meta-analysis: Prevalence of Posttraumatic Stress Disorder in Trauma-Exposed Preschool-Aged Children
Source: J Am Acad Child Adolesc Psychiatry. 2022 Mar;61(3):366–77. doi: 10.1016/j.jaac.2021.05.026 (PMC8885427; doi:10.1016/j.jaac.2021.05.026)
Supplement: Supplement 2 [file mmc2.docx]

**Supplement 2**

**Reference list of excluded articles and justifications**

**Articles in which full texts were examined and then excluded from the meta-analysis (k=119)**

Subheadings detailing the reason for exclusion are provided

**Does not meet age criteria (*k*=30)**

Almqvist, K., & Broberg, A. G. (1999). Mental health and social adjustment in young refugee children y 3½ years after their arrival in Sweden. *Journal of the American Academy of Child & Adolescent Psychiatry*, *38*(6), 723-730. <https://doi.org/10.1097/00004583-199906000-00020>

Deblinger, E., Taub, B., Maedel, A. B., Lippmann, J., & Stauffer, L. B. (1998). Psychosocial factors predicting parent reported symptomatology in sexually abused children. *Journal of Child Sexual Abuse*, *6*(4), 35-49. <https://doi.org/10.1300/j070v06n04_03>

Ellis, A., Stores, G., & Mayou, R. (1998). Psychological consequences of road traffic accidents in children. *European child & adolescent psychiatry*, *7*(2), 61-68. <https://doi.org/10.1007/s007870050048>

Endo, T., Shioiri, T., & Someya, T. (2009). Post‐traumatic symptoms among the children and adolescents 2 years after the 2004 Niigata–Chuetsu earthquake in Japan. *Psychiatry and clinical neurosciences*, *63*(2), 253-253. <https://doi.org/10.1111/j.1440-1819.2008.01914.x>

Famularo, R., Kinscherff, R., & Fenton, T. (1992). Psychiatric diagnoses of maltreated children: preliminary findings. *Journal of the American Academy of Child & Adolescent Psychiatry*, *31*(5), 863-867. <https://doi.org/10.1097/00004583-199209000-00013>

Field, T., Seligman, S., Scafidi, F., & Schanberg, S. (1996). Alleviating posttraumatic stress in children following Hurricane Andrew. *Journal of applied developmental psychology*, *17*(1), 37-50. <https://doi.org/10.1016/s0193-3973(96)90004-0>

Fujiwara, T., Yagi, J., Homma, H., Mashiko, H., Nagao, K., & Okuyama, M. (2017). Symptoms of post-traumatic stress disorder among young children 2 years after the Great East Japan Earthquake. *Disaster medicine and public health preparedness*, *11*(2), 207-215. <https://doi.org/10.1017/dmp.2016.101>

Garfin, D. R., Silver, R. C., Gil-Rivas, V., Guzmán, J., Murphy, J. M., Cova, F., ... & Guzmán, M. P. (2014). Children’s reactions to the 2010 Chilean earthquake: The role of trauma exposure, family context, and school-based mental health programming. *Psychological Trauma: Theory, Research, Practice, and Policy*, *6*(5), 563. <https://doi.org/10.1037/a0036584>

Grasso, D., Boonsiri, J., Lipschitz, D., Guyer, A., Houshyar, S., Douglas-Palumberi, H., ... & Kaufman, J. (2009). Posttraumatic stress disorder: The missed diagnosis. *Child Welfare*, *88*(4), 157.

Green, B.L., Korol. M., Grace, M.C., Vary, M.G., Leonard, A.C., Gleser, G.C., & Smitson-Cohen, S. (1991). Children and disaster: Age, gender, and parental effects on PTSD symptoms. *Journal of the American Academy of Child & Adolescent Psychiatry*, *30*(6), 945-951. <https://doi.org/10.1097/00004583-199111000-00012>

Husain, S. A., Allwood, M. A., & Bell, D. J. (2008). The relationship between PTSD symptoms and attention problems in children exposed to the Bosnian war. *Journal of Emotional and Behavioral Disorders*, *16*(1), 52-62. <https://doi.org/10.1177/1063426607310847>

Kessler, R. C., Duncan, G. J., Gennetian, L. A., Katz, L. F., Kling, J. R., Sampson, N. A., ... & Ludwig, J. (2014). Associations of housing mobility interventions for children in high-poverty neighborhoods with subsequent mental disorders during adolescence. *Jama*, *311*(9), 937-947. <https://doi.org/10.1001/jama.2014.607>

Kocijan-Hercigonja, D., Rijavec, M., Jones, W. P., & Remeta, D. (1996). Psychologic problems of children wounded during the war in Croatia. *Nordic Journal of Psychiatry*, *50*(6), 451-456. <https://doi.org/10.3109/08039489609082513>

Lamers-Winkelman, F., Willemen, A. M., & Visser, M. (2012). Adverse childhood experiences of referred children exposed to intimate partner violence: Consequences for their wellbeing. *Child abuse & neglect*, *36*(2), 166-179. <https://doi.org/10.1016/j.chiabu.2011.07.006>

Lehmann, P., & Elliston, E. J. (2001). Traumatic responding in children exposed to domestic violence: A cross-cultural study. *Journal of Ethnic and Cultural Diversity in Social Work*, *10*(4), 81-102. <https://doi.org/10.1300/j051v10n04_05>

McCloskey, L. A., Fernández‐Esquer, M. E., Southwick, K., & Locke, C. (1995). The psychological effects of political and domestic violence on Central American and Mexican immigrant mothers and children. *Journal of Community Psychology*, *23*(2), 95-115. [https://doi.org/10.1002/1520-6629(199504)23:2<95::aid-jcop2290230202>3.0.co;2-a](https://doi.org/10.1002/1520-6629(199504)23:2%3C95::aid-jcop2290230202%3E3.0.co;2-a)

Mennen, F. E. (2004). PTSD symptoms in abused Latino children. *Child and Adolescent Social Work Journal*, *21*(5), 477-493. <https://doi.org/10.1023/b:casw.0000043360.98041.48>

Nader, K., Pynoos, R., Fairbanks, L., & Frederick, C. (1990). Children's PTSD reactions one year after a sniper attack at their school. *The American journal of psychiatry*. <https://doi.org/10.1176/ajp.147.11.1526>

Peters, V., Sottiaux, M., Appelboom, J., & Kahn, A. (2004). Posttraumatic stress disorder after dog bites in children. *The Journal of pediatrics*, *144*(1), 121-122. <https://doi.org/10.1016/j.jpeds.2003.10.024>

Şahin, N. H., Batıgün, A. D., & Yılmaz, B. (2007). Psychological symptoms of Turkish children and adolescents after the 1999 earthquake: Exposure, gender, location, and time duration. *Journal of Traumatic Stress: Official Publication of The International Society for Traumatic Stress Studies*, *20*(3), 335-345. <https://doi.org/10.1002/jts.20217>

Saltzman, K. M., Weems, C. F., & Carrion, V. G. (2006). IQ and posttraumatic stress symptoms in children exposed to interpersonal violence. *Child Psychiatry and Human Development*, *36*(3), 261-272. <https://doi.org/10.1007/s10578-005-0002-5>

Santiago, C. D., Raviv, T., Ros, A. M., Brewer, S. K., Distel, L. M., Torres, S. A., ... & Langley, A. K. (2018). Implementing the Bounce Back trauma intervention in urban elementary schools: A real-world replication trial. *School Psychology Quarterly*, *33*(1), 1-9. <https://doi.org/10.1037/spq0000229>

Schilpzand, E. J., Sciberras, E., Alisic, E., Efron, D., Hazell, P., Jongeling, B., ... & Nicholson, J. M. (2018). Trauma exposure in children with and without ADHD: prevalence and functional impairment in a community-based study of 6–8-year-old Australian children. *European child & adolescent psychiatry*, *27*(6), 811-819. <https://doi.org/10.1007/s00787-017-1067-y>

Shears, D., Nadel, S., Gledhill, J., Gordon, F., & Garralda, M. E. (2007). Psychiatric adjustment in the year after meningococcal disease in childhood. Journal of the American Academy of Child & Adolescent Psychiatry, 46(1), 76-82. <https://doi.org/10.1097/01.chi.0000242234.83140.56>

Sim, L., Friedrich, W. N., Davies, W. H., Trentham, B., Lengua, L., & Pithers, W. (2005). The Child Behavior Checklist as an indicator of posttraumatic stress disorder and dissociation in normative, psychiatric, and sexually abused children. *Journal of Traumatic Stress: Official Publication of The International Society for Traumatic Stress Studies*, *18*(6), 697-705. <https://doi.org/10.1002/jts.20078>

Soykoek, S., Mall, V., Nehring, I., Henningsen, P., & Aberl, S. (2017). Post-traumatic stress disorder in Syrian children of a German refugee camp. *The Lancet*, *389*(10072), 903-904. <https://doi.org/10.1016/s0140-6736(17)30595-0>

Vaage, A. B., Garløv, I., Hauff, E., & Thomsen, P. H. (2007). Psychiatric symptoms and service utilization among refugee children referred to a child psychiatry department: a retrospective comparative case note study. *Transcultural psychiatry*, *44*(3), 440-458. <https://doi.org/10.1177/1363461507081641>

Wherry, J. N., & Marrs, A. S. (2008). Anxious school refusers and symptoms of PTSD in abused children. *Journal of Child & Adolescent Trauma*, *1*(2), 109-117. <https://doi.org/10.1080/19361520802084061>

Wiguna, T., Guerrero, A. P., Kaligis, F., & Khamelia, M. (2010). Psychiatric morbidity among children in North Aceh district (Indonesia) exposed to the 26 December 2004 tsunami. *Asia‐Pacific Psychiatry*, *2*(3), 151-155. <https://doi.org/10.1111/j.1758-5872.2010.00079.x>

Wu, F., Meng, W. Y., Hao, C. Z., Zhu, L. L., Chen, D. Q., Lin, L. Y., & Wen, H. (2016). Analysis of posttraumatic stress disorder in children with road traffic injury in Wenzhou, China. *Traffic injury prevention*, *17*(2), 159-163. <https://doi.org/10.1080/15389588.2015.1050719>

**Does not meet study design criteria (*k*=4)**

Abd-Elshafy, S. K., Khalaf, G. S., Abo-Kerisha, M. Z., Ahmed, N. T., El-Aziz, M. A. A., & Mohamed, M. A. (2015). Not all sounds have negative effects on children undergoing cardiac surgery. *Journal of cardiothoracic and vascular anesthesia*, *29*(5), 1277-1284. <https://doi.org/10.1053/j.jvca.2015.01.005>

Briggs‐Gowan, M. J., Grasso, D., Bar‐Haim, Y., Voss, J., McCarthy, K. J., Pine, D. S., & Wakschlag, L. S. (2016). Attention bias in the developmental unfolding of post‐traumatic stress symptoms in young children at risk. *Journal of child psychology and psychiatry*, *57*(9), 1083-1091. <https://doi.org/10.1111/jcpp.12577>

Horn, S. R., Miller-Graff, L. E., Galano, M. M., & Graham-Bermann, S. A. (2017). Posttraumatic stress disorder in children exposed to intimate partner violence: the clinical picture of physiological arousal symptoms. *Child care in practice*, *23*(1), 90-103. <https://doi.org/10.1080/13575279.2015.1126229>

Scheeringa, M. S. (2008). Developmental considerations for diagnosing PTSD and acute stress disorder in preschool and school-age children. *The American Journal of Psychiatry, 165 (10),* 1237-1239. <https://doi.org/10.1176/appi.ajp.2008.08070974>

**Indirect trauma (k=2)**

Abbo, C., Kinyanda, E., Kizza, R. B., Levin, J., Ndyanabangi, S., & Stein, D. J. (2013). Prevalence, comorbidity and predictors of anxiety disorders in children and adolescents in rural north-eastern Uganda. *Child and adolescent psychiatry and mental health*, *7*(1), 21. <https://doi.org/10.1186/1753-2000-7-21>

Saylor, C. F., Cowart, B. L., Lipovsky, J. A., Jackson, C., & Finch Jr, A. J. (2003). Media exposure to September 11: Elementary school students' experiences and posttraumatic symptoms. *American Behavioral Scientist*, *46*(12), 1622-1642. <https://doi.org/10.1177/0002764203254619>

**Not measuring pre-school PTSD (*k*=12)**

Al-Jawadi, A. A., & Abdul-Rhman, S. (2007). Prevalence of childhood and early adolescence mental disorders among children attending primary health care centers in Mosul, Iraq: a cross-sectional study. *BMC public health*, *7*(1), 274. <https://doi.org/10.1186/1471-2458-7-274>

Briere, J., Johnson, K., Bissada, A., Damon, L., Crouch, J., Gil, E., ... & Ernst, V. (2001). The Trauma Symptom Checklist for Young Children (TSCYC): Reliability and association with abuse exposure in a multi-site study. *Child abuse & neglect*, *25*(8), 1001-1014. <https://doi.org/10.1016/s0145-2134(01)00253-8>

De Young, A. C., Hendrikz, J., Kenardy, J. A., Cobham, V. E., & Kimble, R. M. (2014). Prospective evaluation of parent distress following pediatric burns and identification of risk factors for young child and parent posttraumatic stress disorder. *Journal of Child and Adolescent Psychopharmacology*, *24*(1), 9-17. <https://doi.org/10.1089/cap.2013.0066>

Endo, T., Shioiri, T., Someya, T., Toyabe, S., & Akazawa, K. (2007). Parental mental health affects behavioral changes in children following a devastating disaster: a community survey after the 2004 Niigata-Chuetsu earthquake. *General hospital psychiatry*, *29(2),* 175-176. <https://doi.org/10.1016/j.genhosppsych.2006.09.006>

Lieberman, A. F., Van Horn, P., & Ippen, C. G. (2005). Toward evidence-based treatment: Child-parent psychotherapy with preschoolers exposed to marital violence. *Journal of the American Academy of Child & Adolescent Psychiatry*, *44*(12), 1241-1248. <https://doi.org/10.1097/01.chi.0000181047.59702.58>

McCrae, J. S. (2009). Emotional and behavioral problems reported in child welfare over 3 years. *Journal of Emotional and Behavioral Disorders*, *17*(1), 17-28. <https://doi.org/10.1177/1063426608319141>

Milburn, N. L., Lynch, M., & Jackson, J. (2008). Early identification of mental health needs for children in care: A therapeutic assessment programme for statutory clients of child protection. *Clinical child psychology and psychiatry*, *13*(1), 31-47. <https://doi.org/10.1177/1359104507086339>

Schrag, N. M., McKeown, R. E., Jackson, K. L., Cuffe, S. P., & Neuberg, R. W. (2008). Stress‐related mental disorders in childhood cancer survivors. *Pediatric blood & cancer*, *50*(1), 98-103. <https://doi.org/10.1002/pbc.21285>

Spence, S. H., Rapee, R., McDonald, C., & Ingram, M. (2001). The structure of anxiety symptoms among preschoolers. *Behaviour research and therapy*, *39*(11), 1293-1316. <https://doi.org/10.1016/s0005-7967(00)00098-x>

Sprang, G., Staton‐Tindall, M., & Clark, J. (2008). Trauma exposure and the drug endangered child. *Journal of Traumatic Stress: Official Publication of The International Society for Traumatic Stress Studies*, *21*(3), 333-339. <https://doi.org/10.1002/jts.20330>

Stoddard, F. J., Saxe, G., Ronfeldt, H., Drake, J. E., Burns, J., Edgren, C., & Sheridan, R. (2006). Acute stress symptoms in young children with burns. *Journal of the American Academy of Child & Adolescent Psychiatry*, *45*(1), 87-93. <https://doi.org/10.1097/01.chi.0000184934.71917.3a>

Stoddard Jr, F. J., Sorrentino, E. A., Ceranoglu, T. A., Saxe, G., Murphy, J. M., Drake, J. E., ... & Sheridan, R. L. (2009). Preliminary evidence for the effects of morphine on posttraumatic stress disorder symptoms in one-to four-year-olds with burns. *Journal of Burn Care & Research*, *30*(5), 836-843. <https://doi.org/10.1097/bcr.0b013e3181b48102>

**Clinical or PTSD sample (*k*=17)**

Als, L. C., Nadel, S., Cooper, M., Vickers, B., & Garralda, M. E. (2015). A supported psychoeducational intervention to improve family mental health following discharge from paediatric intensive care: feasibility and pilot randomised controlled trial. *BMJ open*, *5*(12), e009581. <https://doi.org/10.1136/bmjopen-2015-009581>

De Bellis, M. D., Hooper, S. R., Spratt, E. G., & Woolley, D. P. (2009). Neuropsychological findings in childhood neglect and their relationships to pediatric PTSD. *Journal of the International Neuropsychological Society*, *15*(6), 868-878. <https://doi.org/10.1017/s1355617709990464>

Deblinger, E., Mannarino, A. P., Cohen, J. A., Runyon, M. K., & Steer, R. A. (2011). Trauma‐focused cognitive behavioral therapy for children: impact of the trauma narrative and treatment length. *Depression and anxiety*, *28*(1), 67-75. <https://doi.org/10.1002/da.20744>

Dehon, C., & Scheeringa, M. S. (2006). Screening for preschool posttraumatic stress disorder with the Child Behavior Checklist. *Journal of Pediatric Psychology*, *31*(4), 431-435. <https://doi.org/10.1093/jpepsy/jsj006>

Hulette, A. C., Freyd, J. J., Pears, K. C., Kim, H. K., Fisher, P. A., & Becker-Blease, K. A. (2008). Dissociation and posttraumatic symptoms in maltreated preschool children. *Journal of child & adolescent trauma*, *1*(2), 93-108. <https://doi.org/10.1080/19361520802083980>

Ippen, C. G., Harris, W. W., Van Horn, P., & Lieberman, A. F. (2011). Traumatic and stressful events in early childhood: Can treatment help those at highest risk?. *Child abuse & neglect*, *35*(7), 504-513. <https://doi.org/10.1016/j.chiabu.2011.03.009>

Karacetin, G., Demir, T., Baghaki, S., Cetinkale, O., & Elagoz Yuksel, M. (2014). Psychiatric disorders and their association with burn-related factors in children with burn injury. *Turkish Journal of Trauma and Emergency Surgery*, *20*(3), 176-180. https://doi.org/[10.5505/tjtes.2014.49033](https://dx.doi.org/10.5505/tjtes.2014.49033)

Kiser, L. J., Ackerman, B. J., Brown, E., Edwards, N. B., McColgan, E. D. G. A. R., Pugh, R., & Pruitt, D. B. (1988). Post-traumatic stress disorder in young children: A reaction to purported sexual abuse. *Journal of the American Academy of Child & Adolescent Psychiatry*, *27*(5), 645-649. <https://doi.org/10.1097/00004583-198809000-00023>

Kramer, D. N., & Landolt, M. A. (2014). Early psychological intervention in accidentally injured children ages 2–16: A randomized controlled trial. *European journal of psychotraumatology*, *5*(1), 24402. <https://doi.org/10.3402/ejpt.v5.24402>

Kramer, D. N., Hertli, M. B., & Landolt, M. A. (2013). Evaluation of an early risk screener for PTSD in preschool children after accidental injury. *Pediatrics*, *132*(4), e945-e951. <https://doi.org/10.1542/peds.2013-0713>

Malmquist, C. P. (1986). Children who witness parental murder: Posttraumatic aspects. *Journal of the American Academy of Child Psychiatry*, *25*(3), 320-325. <https://doi.org/10.1016/s0002-7138(09)60253-3>

Scheeringa, M. S., Peebles, C. D., Cook, C. A., & Zeanah, C. H. (2001). Toward establishing procedural, criterion, and discriminant validity for PTSD in early childhood. *Journal of the American Academy of Child & Adolescent Psychiatry*, *40*(1), 52-60. <https://doi.org/10.1097/00004583-200101000-00016>

Scheeringa, M. S., Zeanah, C. H., Myers, L., & Putnam, F. W. (2003). New findings on alternative criteria for PTSD in preschool children. *Journal of the American Academy of Child & Adolescent Psychiatry*, *42*(5), 561-570. <https://doi.org/10.1097/01.chi.0000046822.95464.14>

Scheeringa, M. S., Zeanah, C. H., Myers, L., & Putnam, F. W. (2005). Predictive validity in a prospective follow-up of PTSD in preschool children. *Journal of the American Academy of Child & Adolescent Psychiatry*, *44*(9), 899-906. <https://doi.org/10.1097/01.chi.0000169013.81536.71>

Scheeringa, M. S., Myers, L., Putnam, F. W., & Zeanah, C. H. (2015). Maternal factors as moderators or mediators of PTSD symptoms in very young children: a two-year prospective study. *Journal of Family Violence*, *30*(5), 633-642. <https://doi.org/10.1007/s10896-015-9695-9>

Seng, J. S., Graham-Bermann, S. A., Clark, M. K., McCarthy, A. M., & Ronis, D. L. (2005). Posttraumatic stress disorder and physical comorbidity among female children and adolescents: results from service-use data. *Pediatrics*, *116*(6), e767-e776. <https://doi.org/10.1542/peds.2005-0608>

Smith, T. J., Lindsey, R. A., Bohora, S., & Silovsky, J. F. (2019). Predictors of intrusive sexual behaviors in preschool-aged children. *The Journal of Sex Research*, *56*(2), 229-238. <https://doi.org/10.1080/00224499.2018.1447639>

**Studies duplicating same data sample (*k*=6)**

Cohen, E., Chazan, S., Lerner, M., & Maimon, E. (2010). Posttraumatic play in young children exposed to terrorism: An empirical study. *Infant Mental Health Journal: Official Publication of the World Association for Infant Mental Health*, *31*(2), 159-181. <https://doi.org/10.1002/imhj.20250>

De Young, A. C., Kenardy, J. A., Cobham, V. E., & Kimble, R. (2012). Prevalence, comorbidity and course of trauma reactions in young burn‐injured children. *Journal of Child Psychology and Psychiatry*, *53*(1), 56-63. <https://doi.org/10.1111/j.1469-7610.2011.02431.x>

Meiser-Stedman, R., Smith, P., Yule, W., Glucksman, E., & Dalgleish, T. (2017). Posttraumatic stress disorder in young children three years post-trauma: Prevalence and longitudinal predictors. *The Journal of clinical psychiatry*, *78*(3), 334-339. <https://doi.org/10.4088/jcp.15m10002>

Scheeringa, M. S., Zeanah, C. H., Myers, L., & Putnam, F. W. (2003). New findings on alternative criteria for PTSD in preschool children. *Journal of the American Academy of Child & Adolescent Psychiatry*, *42*(5), 561-570. <https://doi.org/10.1097/01.CHI.0000046822.95464.14>

Scheeringa, M. S., & Zeanah, C. H. (2008). Reconsideration of harm's way: Onsets and comorbidity patterns of disorders in preschool children and their caregivers following Hurricane Katrina. *Journal of Clinical Child & Adolescent Psychology*, *37*(3), 508-518. <https://doi.org/10.1080/15374410802148178>

Scheeringa, M. S. (2019). Development of a brief screen for symptoms of posttraumatic stress disorder in young children: the Young Child PTSD Screen. *Journal of Developmental & Behavioral Pediatrics*, *40*(2), 105-111. <https://doi.org/10.1097/dbp.0000000000000639>

**Data does not describe prevalence (*k*=4)**

DeVoe, E. R., Klein, T. P., Bannon Jr, W., & Miranda-Julian, C. (2011). Young children in the aftermath of the World Trade Center attacks. *Psychological Trauma: Theory, Research, Practice, and Policy*, *3*(1), 1-11. <https://doi.org/10.1037/a0020567>

Drake, J. E., Stoddard Jr, F. J., Murphy, J. M., Ronfeldt, H., Snidman, N., Kagan, J., ... & Sheridan, R. (2006). Trauma severity influences acute stress in young burned children. *Journal of burn care & research*, *27*(2), 174-182. <https://doi.org/10.1097/01.bcr.0000202618.51001.69>

Grasso, D. J., Ford, J. D., & Briggs-Gowan, M. J. (2013). Early life trauma exposure and stress sensitivity in young children. *Journal of pediatric psychology*, *38*(1), 94-103. <https://doi.org/10.1093/jpepsy/jss101>

Stoddard, F. J., Ronfeldt, H., Kagan, J., Drake, J. E., Snidman, N., Murphy, J. M., ... & Sheridan, R. L. (2006). Young burned children: the course of acute stress and physiological and behavioral responses. *American journal of psychiatry*, *163*(6), 1084-1090. <https://doi.org/10.1176/ajp.2006.163.6.1084>

**Questionnaire based (*k*=39)**

Als, L. C., Picouto, M. D., O’Donnell, K. J., Nadel, S., Cooper, M., Pierce, C. M., ... & Garralda, M. E. (2017). Stress hormones and posttraumatic stress symptoms following paediatric critical illness: an exploratory study. *European child & adolescent psychiatry*, *26*(5), 511-519. <https://doi.org/10.1007/s00787-016-0933-3>

Ari, A. B., Peri, T., Margalit, D., Galili-Weisstub, E., Udassin, R., & Benarroch, F. (2018). Surgical procedures and pediatric medical traumatic stress (PMTS) syndrome: Assessment and future directions. *Journal of pediatric surgery*, *53*(8), 1526-1531. <https://doi.org/10.1016/j.jpedsurg.2017.10.043>

Azarian, A., Miller, T. W., & Skriptchenko-Gregorian, V. (1996). Baseline assessment of children traumatized by the Armenian earthquake. *Child psychiatry and human development*, *27*(1), 29-41. <https://doi.org/10.1007/bf02353444>

Bogat, G. A., DeJonghe, E., Levendosky, A. A., Davidson, W. S., & von Eye, A. (2006). Trauma symptoms among infants exposed to intimate partner violence. *Child abuse & neglect*, *30*(2), 109-125. <https://doi.org/10.1016/j.chiabu.2005.09.002>

Breton, J. J., Valla, J. P., & Lambert, J. (1993). Industrial disaster and mental health of children and their parents. *Journal of the American Academy of Child & Adolescent Psychiatry*, *32*(2), 438-445. <https://doi.org/10.1097/00004583-199303000-00028>

Broekman, B. F., Olff, M., Tan, F. M., Schreuder, B. J., Fokkens, W., & Boer, F. (2010). The psychological impact of an adenoidectomy and adenotonsillectomy on young children. *International journal of pediatric otorhinolaryngology*, *74*(1), 37-42. <https://doi.org/10.1016/j.ijporl.2009.10.005>

Chae, Y., Goodman, G. S., Eisen, M. L., & Qin, J. (2011). Event memory and suggestibility in abused and neglected children: Trauma-related psychopathology and cognitive functioning. *Journal of experimental child psychology*, *110*(4), 520-538. <https://doi.org/10.1016/j.jecp.2011.05.006>

Fairbrother, G., Stuber, J., Galea, S., Fleischman, A. R., & Pfefferbaum, B. (2003). Posttraumatic stress reactions in New York City children after the September 11, 2001, terrorist attacks. *Ambulatory Pediatrics*, *3*(6), 304-311 [https://doi.org/10.1367/1539-4409(2003)003<0304:psriny>2.0.co;2](https://doi.org/10.1367/1539-4409(2003)003%3C0304:psriny%3E2.0.co;2)

Fujiwara, T., Mizuki, R., Miki, T., & Chemtob, C. (2015). Association between facial expression and PTSD symptoms among young children exposed to the Great East Japan Earthquake: a pilot study. *Frontiers in psychology*, *6*, 1534. <https://doi.org/10.3389/fpsyg.2015.01534>

Garralda, M. E., Gledhill, J., Nadel, S., Neasham, D., O'Connor, M., & Shears, D. (2009). Longer-term psychiatric adjustment of children and parents after meningococcal disease. *Pediatric Critical Care Medicine*, *10*(6), 675-680. <https://doi.org/10.1097/pcc.0b013e3181ae785a>

Gil‐Rivas, V., & Kilmer, R. P. (2013). Children's adjustment following Hurricane Katrina: The role of primary caregivers. *American journal of orthopsychiatry*, *83*(2-3), 413-421. <https://doi.org/10.1111/ajop.12016>

Hagan, M. J., Sulik, M. J., & Lieberman, A. F. (2016). Traumatic life events and psychopathology in a high risk, ethnically diverse sample of young children: A person-centered approach. *Journal of abnormal child psychology*, *44*(5), 833-844. <https://doi.org/10.1007/s10802-015-0078-8>

Hickman, L. J., Jaycox, L. H., Setodji, C. M., Kofner, A., Schultz, D., Barnes-Proby, D., & Harris, R. (2013). How much does “how much” matter? Assessing the relationship between children’s lifetime exposure to violence and trauma symptoms, behavior problems, and parenting stress. *Journal of interpersonal violence*, *28*(6), 1338-1362. <https://doi.org/10.1177/0886260512468239>

Jonkman, C. S., Schuengel, C., Lindeboom, R., Oosterman, M., Boer, F., & Lindauer, R. J. (2013). The effectiveness of Multidimensional Treatment Foster Care for Preschoolers (MTFC-P) for young children with severe behavioral disturbances: study protocol for a randomized controlled trial. *Trials*, *14*(1), 197. <https://doi.org/10.1186/1745-6215-14-197>

Kaplow, J. B., Howell, K. H., & Layne, C. M. (2014). Do circumstances of the death matter? Identifying socioenvironmental risks for grief‐related psychopathology in bereaved youth. *Journal of Traumatic Stress*, *27*(1), 42-49. <https://doi.org/10.1002/jts.21877>

Kaufman‐Shriqui, V., Werbeloff, N., Faroy, M., Meiri, G., Shahar, D. R., Fraser, D., ... & Pietrzak, R. H. (2013). Posttraumatic stress disorder among preschoolers exposed to ongoing missile attacks in the Gaza war. *Depression and anxiety*, *30*(5), 425-431. <https://doi.org/10.1002/da.22121>

Kisiel, C. L., Fehrenbach, T., Torgersen, E., Stolbach, B., McClelland, G., Griffin, G., & Burkman, K. (2014). Constellations of interpersonal trauma and symptoms in child welfare: Implications for a developmental trauma framework. *Journal of Family Violence*, *29*(1), 1-14. <https://doi.org/10.1007/s10896-013-9559-0>

Laor, N., Wolmer, L., Mayes, L. C., Gershon, A., Weizman, R., & Cohen, D. J. (1997). Israeli preschool children under Scuds: a 30-month follow-up. *Journal of the American Academy of Child & Adolescent Psychiatry*, *36*(3), 349-356. <https://doi.org/10.1097/00004583-199703000-00013>

Lester, P., Stein, J. A., Saltzman, W., Woodward, K., MacDermid, S. W., Milburn, N., ... & Beardslee, W. (2013). Psychological health of military children: Longitudinal evaluation of a family-centered prevention program to enhance family resilience. *Military medicine*, *178*(8), 838-845. <https://doi.org/10.7205/milmed-d-12-00502>

Levendosky, A. A., Huth-Bocks, A. C., Semel, M. A., & Shapiro, D. L. (2002). Trauma symptoms in preschool-age children exposed to domestic violence. *Journal of interpersonal Violence*, *17*(2), 150-164. <https://doi.org/10.1177/0886260502017002003>

Magwaza, A. S., Killian, B. J., Petersen, I., & Pillay, Y. (1993). The effects of chronic violence on preschool children living in South African townships. *Child Abuse & Neglect*, *17*(6), 795-803. <https://doi.org/10.1016/s0145-2134(08)80010-5>

Milot, T., Éthier, L. S., St-Laurent, D., & Provost, M. A. (2010). The role of trauma symptoms in the development of behavioral problems in maltreated preschoolers. *Child abuse & neglect*, *34*(4), 225-234. <https://doi.org/10.1016/j.chiabu.2009.07.006>

Mongillo, E. A., Briggs-Gowan, M., Ford, J. D., & Carter, A. S. (2009). Impact of traumatic life events in a community sample of toddlers. *Journal of Abnormal Child Psychology*, *37*(4), 455-468. <https://doi.org/10.1007/s10802-008-9283-z>

Nader, K., Stuber, M., & Pynoos, R. (1991). Posttraumatic stress reactions in preschool children with catastrophic illness: Assessment needs. *Comprehensive Mental Health Care, 1(3),* 223-239.

Nilsson, D., Gustafsson, P. E., & Svedin, C. G. (2012). The psychometric properties of the Trauma Symptom Checklist for Young Children in a sample of Swedish children. *European journal of psychotraumatology*, *3*(1), 18505. <https://doi.org/10.3402/ejpt.v3i0.18505>

Nuttman-Shwartz, O. (2017). Children and adolescents facing a continuous security threat: Aggressive behavior and post-traumatic stress symptoms. *Child Abuse & Neglect*, *69*, 29-39. <https://doi.org/10.1016/j.chiabu.2017.04.008>

Pernebo, K., Fridell, M., & Almqvist, K. (2018). Outcomes of psychotherapeutic and psychoeducative group interventions for children exposed to intimate partner violence. *Child abuse & neglect*, *79*, 213-223. <https://doi.org/10.1016/j.chiabu.2018.02.014>

Piyasil, V., Ketumarn, P., Prubrukarn, R., Pacharakaew, S., Dumrongphol, H., Rungsri, S., ... & Theerawongseree, S. (2008). Psychiatric disorders in children at one year after the tsunami disaster in Thailand. *J Med Assoc Thai*, *91*(Suppl 3), S15-S20.

Rivara, F. P., McCarty, C. A., Shandro, J., Wang, J., & Zatzick, D. (2014). Parental injury and psychological health of children. *Pediatrics*, *134*(1), e88-e97. <https://doi.org/10.1542/peds.2013-3273>

Rossman, B. R., Bingham, R. D., & Emde, R. N. (1997). Symptomatology and adaptive functioning for children exposed to normative stressors, dog attack, and parental violence. *Journal of the American Academy of Child & Adolescent Psychiatry*, *36*(8), 1089-1097. <https://doi.org/10.1097/00004583-199708000-00016>

Sadeh, A., Hen-Gal, S., & Tikotzky, L. (2008). Young children's reactions to war-related stress: A survey and assessment of an innovative intervention. *Pediatrics*, *121*(1), 46-53. <https://doi.org/10.1542/peds.2007-1348>

Saigh, P. A., Yasik, A. E., Mitchell, P., & Abright, A. R. (2011). The psychological adjustment of a sample of New York City preschool children 8–10 months after September 11, 2001. *Psychological Trauma: Theory, Research, Practice, and Policy*, *3*(2), 109-116. <https://doi.org/10.1037/a0020701>

Schechter, D. S., Zygmunt, A., Coates, S. W., Davies, M., Trabka, K. A., McCaw, J., ... & Robinson, J. L. (2007). Caregiver traumatization adversely impacts young children's mental representations on the MacArthur Story Stem Battery. *Attachment & Human Development*, *9*(3), 187-205. <https://doi.org/10.1080/14616730701453762>

Schultz, D., Jaycox, L. H., Hickman, L. J., Setodji, C., Kofner, A., Harris, R., & Barnes, D. (2013). The relationship between protective factors and outcomes for children exposed to violence. *Violence and victims*, *28*(4), 697-714. <https://doi.org/10.1891/0886-6708.vv-d-12-00005>

Shehadeh, A., Loots, G., Vanderfaeillie, J., & Derluyn, I. (2015). The impact of parental detention on the psychological wellbeing of Palestinian children. *PloS one*, *10*(7), e0133347. <https://doi.org/10.1371/journal.pone.0133347>

Stuber, M. L., Nader, K., Yasuda, P., Pynoos, R. S., & Cohen, S. (1991). Stress responses after pediatric bone marrow transplantation: Preliminary results of a prospective longitudinal study. *Journal of the American Academy of Child & Adolescent Psychiatry*, *30*(6), 952-957. <https://doi.org/10.1097/00004583-199111000-00013>

Vasileva, M., Haag, A. C., Landolt, M. A., & Petermann, F. (2018). Posttraumatic Stress Disorder in Very Young Children: Diagnostic Agreement Between ICD‐11 and DSM‐5. *Journal of traumatic stress*, *31*(4), 529-539. <https://doi.org/10.1002/jts.22314>

Vasileva, M., & Petermann, F. (2017). Posttraumatic stress symptoms in preschool children in foster care: the influence of placement and foster family environment. *Journal of traumatic stress*, *30*(5), 472-481. <https://doi.org/10.1002/jts.22217>

Yagi, J., Fujiwara, T., Yambe, T., Okuyama, M., Kawachi, I., & Sakai, A. (2016). Does social capital reduce child behavior problems? Results from the Great East Japan Earthquake follow-up for Children Study. *Social psychiatry and psychiatric epidemiology*, *51*(8), 1117-1123. <https://doi.org/10.1007/s00127-016-1227-2>

**Non-standardised interviews (*k*=4)**

Almqvist, K., & Brandell-Forsberg, M. (1997). Refugee children in Sweden: Post-traumatic stress disorder in Iranian preschool children exposed to organized violence. *Child abuse & neglect*, *21*(4), 351-366. <https://doi.org/10.1016/s0145-2134(96)00176-7>

Dawes, A., Tredoux, C., & Feinstein, A. (1989). Political violence in South Africa: Some effects on children of the violent destruction of their community. *International journal of mental health*, *18*(2), 16-43. <https://doi.org/10.1080/00207411.1989.11449122>

Feldman, R., & Vengrober, A. (2011). Posttraumatic stress disorder in infants and young children exposed to war-related trauma. *Journal of the American Academy of Child & Adolescent Psychiatry*, *50*(7), 645-658. <https://doi.org/10.1016/j.jaac.2011.03.001>

Halevi, G., Djalovski, A., Vengrober, A., & Feldman, R. (2016). Risk and resilience trajectories in war‐exposed children across the first decade of life. *Journal of Child Psychology and Psychiatry*, *57*(10), 1183-1193. <https://doi.org/10.1111/jcpp.12622>

**PTSD measure before 1 month (*k*=1)**

Haag, A. C., & Landolt, M. A. (2017). Young children’s acute stress after a burn injury: Disentangling the role of injury severity and parental acute stress. *Journal of pediatric psychology*, *42*(8), 861-870. <https://doi.org/10.1093/jpepsy/jsx059>
