# Supplementary material for: Systematic Review and Meta-analysis: Prevalence of Posttraumatic Stress Disorder in Trauma-Exposed Preschool-Aged Children
Source: J Am Acad Child Adolesc Psychiatry. 2022 Mar;61(3):366–77. doi: 10.1016/j.jaac.2021.05.026 (PMC8885427; doi:10.1016/j.jaac.2021.05.026)
Supplement: Table S1 [file mmc3.docx]

**Individual study outcome of risk of bias assessment**

Table S1: Risk of bias assessment outcomes by criteria
